# Obituary

**Published:** 1912-03-02

**Authors:** 


### Obituary.

Dr. David Christison has died, at the age of eighty-two, at his residence in Edinburgh. He was a son of the well-known Sir Robert Christison, a Professor to the University of Edinburgh. Dr. David Christison graduated at Edinburgh in 1851 as M.D., and after holding a resident appointment at the Royal Infirmary, he went out to the Dardanelles at the time of the Crimean war. He served in the Renkioi Hospital until he contracted a severe illness, and was sent home. After his recovery he gave up the idea of pursuing a medical career, and turned his attention to archæology. In this department the value of his researches is recognised to be immense, and in 1906 the University of Edinburgh conferred upon him the honorary degree of LL.D. Dr. Christison published many important papers, and his work on the growth of trees is scarcely less important than his great contributions to archæology.

Dr. Stanley Lewis Haynes, of Great Malvern, has died, in retirement, at the age of seventy-one. He was formerly Medical Officer of Health for Malvern Link and Surgeon-accoucheur to the Malvern Lying-in Charity. He qualified in 1863 at Edinburgh as M.D., and in London as M.R.C.S. He had considerable experience in lunacy work, holding the appointments of assistant physician at the Royal Asylum, Edinburgh, and medical superintendent at Laverstock House Asylum, in Salisbury. Among his active interests must be recorded that in connection with the Volunteers. Dr. Haynes served a long time in the 1st Worcester Volunteer Artillery, attaining the rank of Surgeon-Major in that corps. Numerous contributions on various aspects of insanity and asylum work were made by Dr. Haynes, and he attended with regularity the meetings of many learned societies, especially those of the Worcester Medical Society, of which he was one of the former Presidents.

Mr. James Taylor, of Chester and Ruabon, has died at Chester, where he was known not only as one of the most prominent surgeons in North Wales but also as one of the most active justices of the peace for Chester. Mr. Taylor had been associated with Chester from the very beginning of his medical career, for he was appointed resident house-surgeon to the infirmary there soon after qualifying in 1864. This he did in London, after studying at Anderson's College, Glasgow, and at Birmingham. Later on he took the fellowship of the Royal College of Surgeons of England, and worked up a very successful surgical practice. He was appointed Honorary Surgeon to the Chester General Infirmary, and in due course became one of its consulting surgeons. He was an active member of many learned societies, and a former president of the local branch of the British Medical Association. His public life in Chester was of the greatest value, and will cause his loss to be severely felt in that city.

